# The C-TERMINUS of AtGRIP Is Crucial for Its Self-Association and for Targeting to Golgi Stacks in *Arabidopsis*

**DOI:** 10.1371/journal.pone.0098963

**Published:** 2014-06-05

**Authors:** Lei Zhao, Yan Li

**Affiliations:** State Key Laboratory of Plant Physiology and Biochemistry, College of Biological Sciences, China Agricultural University, Beijing, China; Institute of Botany, Chinese Academy of Sciences, China

## Abstract

**Background:**

In animals and fungi, dimerization is crucial for targeting GRIP domain proteins to the Golgi apparatus. Only one gene in the *Arabidopsis* genome, *AtGRIP*, codes for a GRIP domain protein. It remains unclear whether AtGRIP has properties similar to those of GRIP domain proteins.

**Results:**

In this study, western blot and yeast two-hybrid analyses indicated that AtGRIPs could form a parallel homodimer. In addition, yeast two-hybrid analysis indicated that AtGRIP_aa711–753_, AtGRIP_aa711–766_ and AtGRIP_aa711–776_ did not interact with themselves, but the intact GRIP domain with the AtGRIP C-terminus did. Confocal microscopy showed that only an intact GRIP domain with an AtGRIP C-terminus could localize to the Golgi stacks in *Arabidopsis* leaf protoplasts. A BLAST analysis showed that the C-terminus of GRIP proteins was conserved in the plant kingdom. Mutagenesis and yeast two-hybrid analyses showed that the L742 of AtGRIP contributed to dimerization and was crucial for Golgi localization.

**Conclusions:**

These results indicate that the C-terminus of GRIP proteins is essential for self-association and for targeting of Golgi stacks in plant cells. We suggest that several properties of GRIP proteins differ between plant and animal cells.

## Introduction

The Golgi apparatus consists of a series of dynamically stacked cisternae and plays a key role in glycoprotein modification and processing along with vesicle classification and secretion in eukaryotic cells. The *trans*-Golgi network (TGN) is a highly dynamic and complicated subcompartment of the Golgi apparatus, and it comprises an extensive membrane network on the *trans* face of the Golgi apparatus that is able to form numerous tubules [1], [2], [3]. Many binding proteins are present in the TGN, and they are most likely associated with structural maintenance in addition to vesicle classification and secretion [4], [5], [6].

A family of peripheral membrane proteins with extensive coiled-coil regions and a C-terminal GRIP domain was recently characterized in the TGN. GRIP domain proteins have been identified in animals, plants, yeasts and protozoa [7], [8], and GRIP domain targeting to the Golgi is conserved in animal cells [9]–[11].

Previous reports indicated that GRIP domain proteins play an important role in maintaining the structural and functional stability of the TGN in animal cells, and alterations in the levels of these proteins could induce structural abnormalities and/or disturb the membrane transport pathway in the TGN [12]–[14]. For instance, over-expression of the golgin-245/p230 GRIP domain induced TGN disruption [14] and inhibited the ability of golgin-97 to block endosome-to-TGN transport [13].

The GRIP domain proteins are effectors of the small GTPase Arl1 [15]–[17]. Active Arl1 (Arl1-GTP) regulates the localization of GRIP domain proteins to the Golgi membrane. The crystal structure of Arl1-GTP in association with the GRIP domain of golgin-245/p230 showed that the GRIP domain can form a homodimer in which each monomer binds one Arl1-GTP. An N-terminal myristyl group anchors Arl1-GTP to the membrane, allowing GRIP domain recruitment to the Golgi [17], [18].

Previous studies showed that at least two interfaces exist in the GRIP domain dimers of golgin-245/p230 and golgin-97, namely the Arl1-GTP/GRIP and the GRIP/GRIP interfaces [17], [18]. Additionally, a third interface has been identified that interacts directly with the membrane [19]. Several conserved amino acids are located at all three interfaces [8], [10], [15], [17], [18], [20]. The highly conserved amino acids located at the Arl1-GTP/GRIP interface, including Y679 in golgin-97 [15] and Y2177 in golgin-245/p230 [17], [18], fit into the Arl1 hydrophobic selectivity pocket, and mutation of this tyrosine to alanine disrupts the interaction with Arl1-GTP and abolishes the Golgi targeting of the mutant protein [17]–[19]. Other conserved amino acids are also located in the GRIP/GRIP interface [17], [18], such as Y2185 and L2202 in golgin-245/p230 [10], [18], [19]. It is crucial that L2202 of one monomer has a strong hydrophobic interaction with Y2185 of another monomer to achieve proper GRIP domain dimerization [18]. Mutations of these crucial amino acids will abolish the formation of GRIP dimers and consequently abolish the Golgi targeting of the GRIP domain [10], [18], [19]. Although the GRIP/GRIP and Arl1-GTP/GRIP interfaces have no overlapping residues in the GRIP domain, the resulting monomeric GRIP domain did not interact with Arl1-GTP in previous studies and did not localize to the Golgi [18], [19].

In the *Arabidopsis* genome, only one gene, *AtGRIP*, codes for a GRIP domain protein [7]. Previous investigations established that the *AtGRIP*-coded protein localizes to the *trans* Golgi and/or TGN [21], [22], [23] and that the mutation of a strictly conserved tyrosine residue (Y717 or K719) to alanine in the GRIP domain abolishes its Golgi targeting [21], [24]. This Y717 amino acid is equivalent to the Y2177 residue in golgin-245/p230, which participates in the interaction between GRIP protein and Arl1. In addition, Stefano et al. also found that AtGRIP could form a dimer [24]. However, whether AtGRIP has properties similar to those of GRIP domain proteins remains to be established.

AtGRIP was shown to form a parallel homodimer in this study. In addition, a yeast two-hybrid analysis indicated that only the intact GRIP domain with the AtGRIP C-terminus could interact with itself and localize to the Golgi stacks. A BLAST analysis showed that the GRIP protein C-terminus was conserved in the plant kingdom. Furthermore, mutagenesis and yeast two-hybrid analyses showed that the L742 of AtGRIP contributed to dimerization and was crucial for Golgi localization.

## Results and Discussion

### AtGRIPs Form a Parallel Homodimer

**Figure 1:**
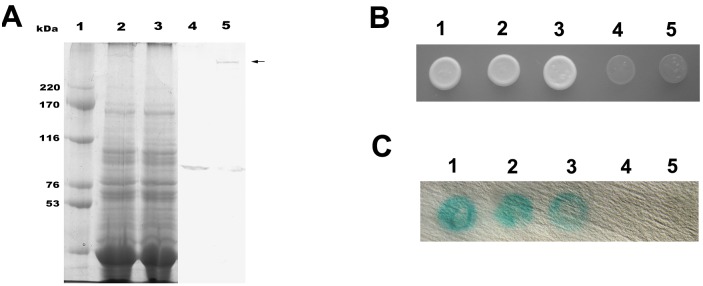
AtGRIP dimerization analysis by western blot and yeast two-hybrid assays. (A) (1) Standard molecular weight marker. (2) SDS-PAGE of *Arabidopsis* protein extract. (3) SDS-PAGE of *Arabidopsis* protein extract treated with 0.25 mM DTSSP. (4) Western blot analysis using the purified anti-AtGRIP antibody showed a 92 kDa band in lane 2. (5) Western blot analysis using the purified anti-AtGRIP antibody showed a band of more than 220 kDa in lane 3 (arrow). (B) The interaction among full-length AtGRIP, AtGRIP (AA1-605) and AtGRIP (AA605–788) was analyzed by using the yeast two-hybrid system. After 7 days on synthetic plates lacking adenine, histidine, leucine and tryptophan at 30°C, only the combinations pGBKT7-AtGRIP with pGADT7-AtGRIP (1), pGBKT7-AtGRIP (AA1-605) with pGADT7-AtGRIP (AA1-605) (2) and pGBKT7-AtGRIP (AA605–788) with pGADT7-AtGRIP (AA605–788) (3) produced colonies. Neither pGBKT7-AtGRIP (AA1-605) with pGADT7-AtGRIP (AA605–788) nor pGBKT7-AtGRIP (AA605–788) with pGADT7- AtGRIP (AA1-605) generated colonies (4, 5). (C) Colonies in (1)–(3) tested positive in the X-gal assay, demonstrating that these fusion proteins can interact. Colonies in (4) and (5) did not test positive in the X-gal assay.

Endogenous *Arabidopsis* AtGRIP was detected as a 92 kDa monomer by western blotting with an anti-AtGRIP antibody [23]. After treatment with the chemical cross-linker 3, 3′-dithiobis (sulphosuccinimidyl propionate) (DTSSP), western blot showed a band of more than 220 kDa in the extract from an *Arabidopsis* seedling (Figure 1A Lane 5). These results indicated that the AtGRIP protein can form a homoligomer.

To determine whether AtGRIP proteins form parallel or anti-parallel homoligomers, the interaction among full-length AtGRIP, AtGRIP_aa1-604_ and AtGRIP_aa605–788_ was analyzed using the yeast two-hybrid system [25]. We found that AtGRIP and its N- and C-terminal fragments can interact with themselves, but the N-terminal fragment cannot interact with the C-terminal fragment (Figure 1B–C). These results suggest that AtGRIP protein can form parallel but not anti-parallel homoligomers.

The GRIP domain proteins can form α-helical parallel homodimers [26], in which each subunit interacts separately with one Arl1-GTP, and the Golgi localization of GRIP domain proteins in animal and yeast cells is dependent on the presence of Arl1 [17], [18]. GRIP domain dimerization is critical for Golgi targeting because the disruption of this dimerization's formation results in the loss of Golgi targeting. Mutagenesis and structural studies have indicated that the dimeric form of the GRIP domain plays structural and functional roles in Golgi targeting in animal cells [17], [18]. ARL1 was also shown to play a role in targeting of the AtGRIP protein to Golgi stacks in plant cells [21], [24]. Stefano et al. found that AtGRIP could form dimers in tobacco leaf epidermal cells [24]. In this study, a yeast two-hybrid analysis indicated that AtGRIPs could form parallel homoligomers. Western blots revealed a band that was larger than 220 kDa, which is more than twice the molecular weight of AtGRIP. However, the apparent size of a cross-linked dimer could be much less than twice the mobility of a monomer. In addition, no intermediate dimers form from partial cross linking. Hence, we believe that AtGRIP may form homodimers, as previously observed for GRIP domain proteins in animal cells.

### The C-terminus of AtGRIP is Crucial for its Self-association and for Targeting to Golgi Stacks

**Figure 2:**
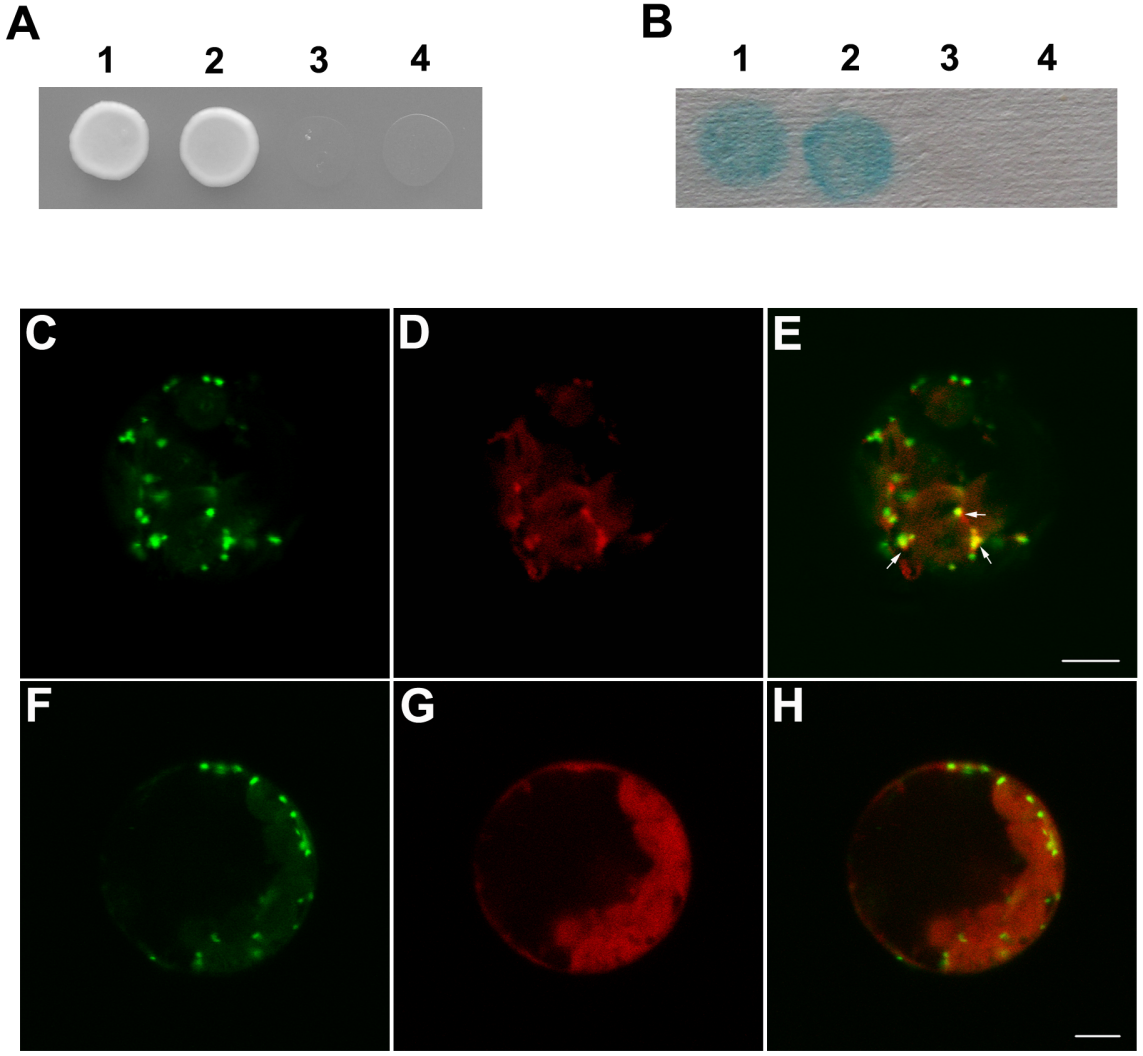
Dimerization analysis of full-length AtGRIP, AtGRIP_aa711_ _–**788**_ **, AtGRIP_aa711_**_–**766**_ **and AtGRIP_aa711_**_–**776**_ **by yeast two-hybrid assays and confocal imaging of** ***Arabidopsis*** **leaf protoplasts that were transiently expressing Nag-GFP concomitantly with AtGRIP_aa711_**_–**788**_ **-mCherry and AtGRIP_aa711_**_–**766**_ **-mCherry.** (A) The interaction among full-length AtGRIP, AtGRIP (AA711–788), AtGRIP (AA711–766) and AtGRIP (AA711–776) was analyzed by using the yeast two-hybrid system. After 7 days on synthetic plates lacking adenine, histidine, leucine and tryptophan at 30°C, only the combinations of pGBKT7-AtGRIP with pGADT7-AtGRIP (1) and pGBKT7-AtGRIP (AA711–788) with pGADT7-AtGRIP (AA711–788) (2) produced colonies. Neither pGBKT7-AtGRIP (AA711–766) with pGADT7-AtGRIP (AA711–766) (3) nor pGBKT7-AtGRIP (AA711–776) with pGADT7-AtGRIP (AA711–776) (4) generated colonies. (B) Colonies in (1) and (2) tested positive in the X-gal assay, demonstrating that these fusion proteins can interact. Colonies in (3) and (4) did not test positive in the X-gal assay. (C), (F) Nag-GFP showed Golgi stacks in the *Arabidopsis* leaf protoplasts. (D) AtGRIP_aa711–788_-mCherry in *Arabidopsis* leaf protoplasts showed punctuated fluorescence. (G) AtGRIP_aa711–766_-mCherry showed disperse fluorescence in *Arabidopsis* leaf protoplasts. (E) Merged image showing the AtGRIP_aa711–788_-mCherry signal pseudo-colored in red and Nag-GFP in green, demonstrating co-localization of AtGRIP_aa711–788_ and the Golgi stacks in the cell (arrows). (H) Merged image showing the AtGRIP_aa711–766_-mCherry signal as pseudo-colored in red and Nag-GFP in green. Bars: 5 µm.

**Figure 3:**
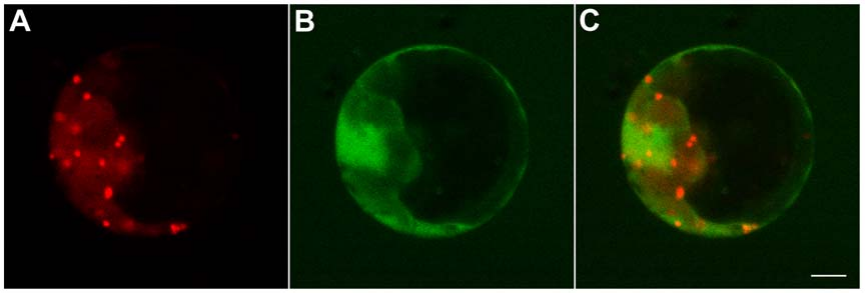
Confocal images of *Arabidopsis* leaf protoplasts transiently expressing Nag-mCherry concomitantly with AtGRIP_aa711–776_-GFP. (A) Nag-mCherry was present in Golgi stacks in *Arabidopsis* leaf protoplasts. (B) AtGRIP_aa711–776_-GFP showed dispersed fluorescence in *Arabidopsis* leaf protoplasts. (C) Merged image showing the Nag-mCherry signal pseudo-colored in red and AtGRIP_aa711–776_-GFP in green. Bar: 5 µm.

The yeast two-hybrid analysis in this study showed that AtGRIP_aa711–753_ could not interact with itself. However, AtGRIP_aa711–788_ interacted with itself (Figure S1). In addition, the yeast two-hybrid analysis also showed that AtGRIP_aa711–766_ and AtGRIP_aa711–776_ could not interact with themselves (Figure 2A–B). Additionally, transient transfection of AtGRIP_aa711–766_ and AtGRIP_aa711–776_ into *Arabidopsis* protoplasts indicated that they could not localize to the Golgi stacks, whereas AtGRIP_aa711–788_ could (Figure 2C–H, Figure 3).

**Figure 4:**
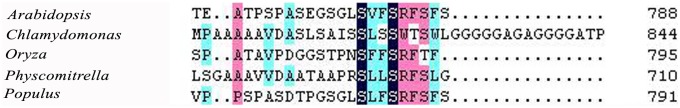
Multiple alignments of the GRIP C-terminus from different organisms, namely *Arabidopsis thaliana*, *Chlamydomonas reinhardtii*, *Oryza sativa*, *Physcomitrella patens* and *Populus trichocarpa*. This image shows that the GRIP C-terminus is conserved in the plant kingdom. The locus of each protein used for NCBI alignment was as follows: *Arabidopsis thaliana* AED98143, *Chlamydomonas reinhardtii* EDP02398, *Oryza sativa* BAC83565, *Physcomitrella patens* EDQ81377 and *Populus trichocarpa* XP_002306424.

We performed BLASTP searches in GenBank by using the AtGRIP amino acid sequence and found that the GRIP protein C-terminus was conserved in the plant kingdom (Figure 4). Therefore, the C-terminal critical residues of the AtGRIP protein may span from S780 to F787.

A previous study showed that the GRIP domain of AtGRIP comprised amino acids 714 to 752 [7]. However, the 45-aa GRIP domain of AtGRIP in *Arabidopsis*, AtGRIP_aa711–755_, did not localize to the Golgi apparatus, whereas AtGRIP_aa605–788_ did [21]. Additionally, the last 77 amino acids of AtGRIP (G712 to S788) were shown to localize to the Golgi apparatus in tobacco cells [7], and the 42-aa GRIP domain of golgin-245/p230 localized to the Golgi apparatus in animal cells [10]. In addition, the C-terminus of GRIP proteins is not conserved in animal cells. Here, we found that AtGRIP_aa711–753_ could not interact with itself. These results could explain why AtGRIP_aa711–755_ did not localize to the Golgi, i.e., because the monomeric GRIP domain could not interact with Arl1-GTP [18], [19]. Further investigation also established that AtGRIP_aa711–766_ and AtGRIP_aa711–776_ could not interact with themselves. However, AtGRIP_aa711–788_, which contains an intact C-terminus, interacted with itself and localized to Golgi stacks. In addition, a BLAST analysis showed that the C-terminus of GRIP proteins was conserved in the plant kingdom. Based on these results, we suggest that the C-terminus of GRIP is essential for its dimerization and for targeting to Golgi stacks in plant cells, which is not consistent with known data on GRIP proteins in animal cells.

### The L742 of AtGRIP Affects the Dimerization and Golgi Stack Targeting of its GRIP Domain

**Figure 5:**
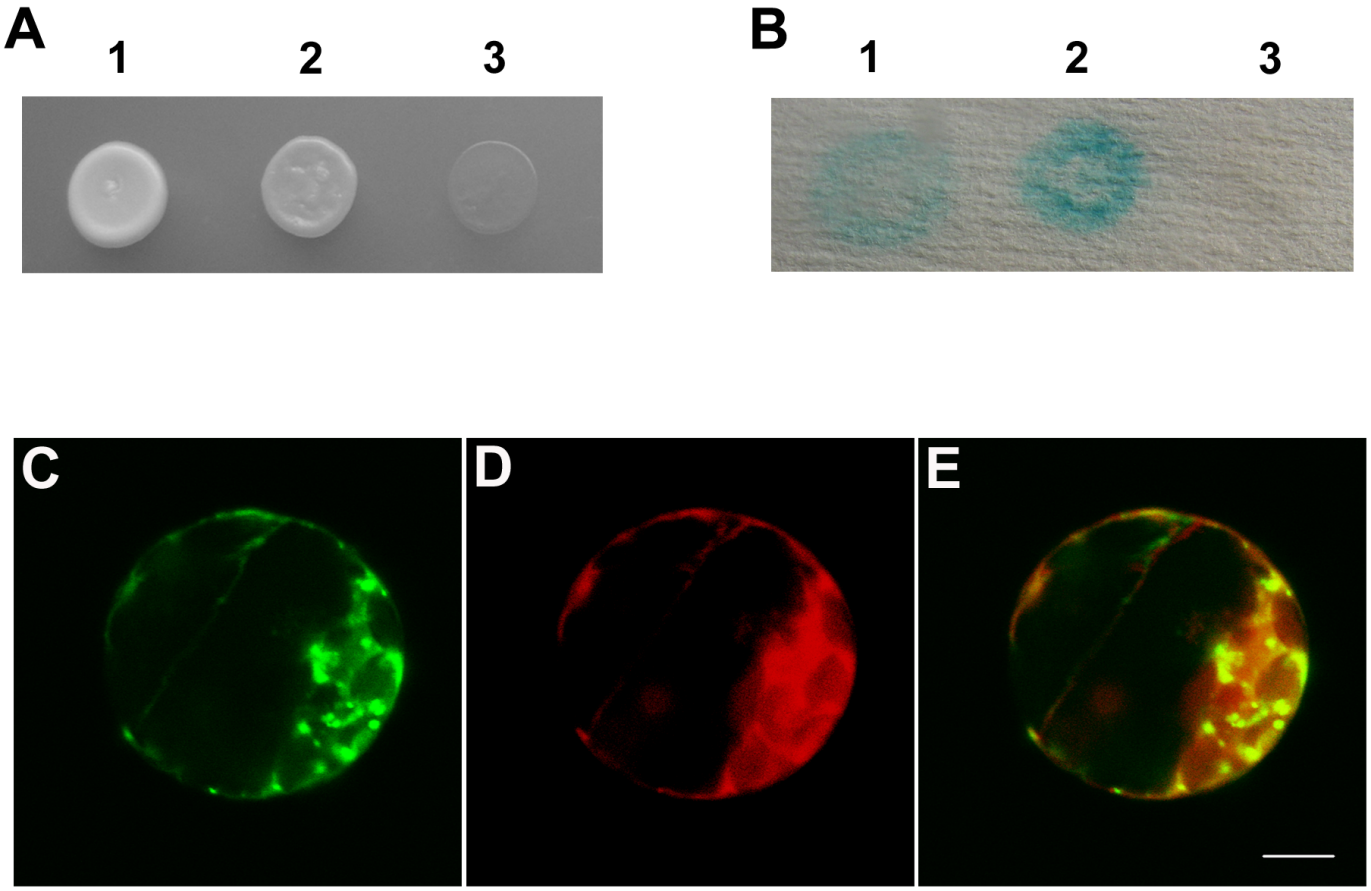
Yeast two-hybrid analysis of AtGRIP_aa711–788_-L742A dimerization and confocal images of *Arabidopsis* leaf protoplasts that were transiently expressing Nag-GFP concomitantly with AtGRIP_aa711–788_-L742A. (A) The interaction among full-length AtGRIP, AtGRIP (AA711–788) and AtGRIP (AA711–788-L742A) was analyzed by using the yeast two-hybrid system. After 7 days on synthetic plates lacking adenine, histidine, leucine and tryptophan at 30°C, only the combinations of pGBKT7-AtGRIP with pGADT7-AtGRIP (1) and pGBKT7-AtGRIP (AA711–788) with pGADT7-AtGRIP (AA711–788) (2) produced colonies. pGBKT7-AtGRIP (AA711–788-L742A) with pGADT7-AtGRIP (AA711–788-L742A) did not generate colonies (3). (B) Colonies in (1) and (2) tested positive in the X-gal assay, demonstrating that these fusion proteins can interact. Colony in (3) did not test positive in the X-gal assay. (C) Nag-GFP showed Golgi stacks in *Arabidopsis* leaf protoplasts. (D) AtGRIP_aa711–788_-L742A-mCherry showed disperse fluorescence in *Arabidopsis* leaf protoplasts. (E) Merged image showing the AtGRIP_aa711–788_-L742A-mCherry signal in pseudo-colored red and Nag-GFP in green. Bar: 5 µm.

The self-interaction of AtGRIP_aa711–788_-L742A was analyzed by using the yeast two-hybrid system, which showed that AtGRIP_aa711–788_-L742A could not interact with itself (Figure 5A–B). However, AtGRIP_aa711–788_ interacted with itself, thereby indicating that L742A is crucial for dimerization of the GRIP domain in AtGRIP.

AtGRIP_aa711–788_-L742A was overexpressed in protoplasts from *Arabidopsis* leaves by transfection and showed a dispersed distribution in the cytoplasm (Figure 5C–E), demonstrating that AtGRIP dimerization is essential for Golgi targeting.

Several pivotal amino acids are distributed among the three interfaces of the GRIP dimer [17]–[19], including the Arl1-GTP/GRIP interface, the GRIP/GRIP interface and the third interface that interacts directly with the membrane [19]. These sequences have all been highly conserved through evolution [8]–[10], [15], [17], [18]. Previous studies have shown that the strictly conserved Y717 and K719 of AtGRIP were crucial for Golgi targeting in *Arabidopsis* [21], [24]. This Y717 amino acid is equivalent to the Y2177 residue in golgin-245/p230 [7], [17], [18], [21]. Because Y2177 is in the Arl1-GTP/GRIP interface, the Y717 of AtGRIP should be important for the interaction between Arl1 and AtGRIP. L742 of AtGRIP is also highly conserved [7], [24], and it is equivalent to the L2202 residue in the GRIP/GRIP interface of golgin-245/p230 [17]–[19]. Mutation of L2202 to alanine in animal cells disrupted the dimerization of the golgin-245/p230 GRIP domain and consequently disrupted its Golgi targeting. Here, we confirmed that the mutation of L742 to alanine disrupted AtGRIP dimerization and consequently abolished its Golgi targeting. These results demonstrate that L742 is crucial for AtGRIP dimerization, and this dimerization is essential for the Golgi targeting of GRIP domain proteins in plant cells.

## Conclusions

AtGRIP was found to form parallel homodimers. We found that only an intact GRIP domain with an AtGRIP C-terminus could interact with itself and localize to the Golgi stacks. In addition, a BLAST analysis showed that the C-terminus of GRIP proteins was conserved in the plant kingdom. These results indicate that C-terminus is essential for the self-association and Golgi targeting of GRIP proteins in plant cells. We therefore suggest that GRIP domain proteins have several different properties between plant and animal cells. Furthermore, mutagenesis and yeast two-hybrid analyses showed that L742 in AtGRIP contributed to dimerization and was crucial for Golgi localization.

## Materials and Methods

### Plant Materials

*Arabidopsis thaliana* plants of the Columbia ecotype were used. *Arabidopsis* seeds were germinated on solid medium containing MS salt and 0.8% agar under short-day conditions (12 h light/12 h dark, 20°C) in Petri dishes.

### Construction of Expression Plasmids

The Nag-GFP plasmid was kindly provided by Dr. Bo Liu.

The AtGRIP fragments, namely AtGRIP_aa1-604_, AtGRIP_aa605–788_, AtGRIP_aa711–753_, AtGRIP_aa711–766_, AtGRIP_aa711–776_, AtGRIP_aa711–788_ and AtGRIP_aa711–788_-L742A, and full-length AtGRIP were amplified by PCR with gene-specific primers. The PCR products were cloned into the pGBKT7 and pGADT7 vectors (Clontech).

A point mutation from L742 to alanine was introduced into AtGRIP_aa711–788_ by standard PCR-mediated mutagenesis.

AtGRIP_aa711–766_, AtGRIP_aa711–788_ and AtGRIP_aa711–788_-L742A were cloned in the transient expression vector pBI221 to generate fusion proteins with mCherry. AtGRIP_aa711–776_ was cloned in the transient expression vector pBI221 to generate fusion proteins with GFP. NAG was cloned in the transient expression vector pBI221 to generate fusion proteins with mCherry.

### SDS-PAGE and Western Blotting

SDS-PAGE was performed according to Laemmli [27], and the resolved products were transferred to a nitrocellulose membrane according to Towbin et al. [28]. The blots were probed with an affinity-purified anti-AtGRIP antibody. The primary antibody was rabbit polyclonal anti-AtGRIP (diluted 1∶150) [23], and the secondary antibody was an alkaline phosphatase-conjugated goat anti-rabbit polyvalent immunoglobulin (Sigma, diluted 1∶60,000). The chromogenic substrates for alkaline phosphatase were nitroblue tetrazolium chloride and 5-bromo-4-chloro-3-indolyl-phosphate (Promega).

### Cross-linking

*Arabidopsis* seedlings were triturated in liquid nitrogen and resuspended in 0.1 M PBS (pH 7.0). The extract was centrifuged twice at 12000 rpm for 10 min each at 4°C, and the supernatant was incubated with 0.05 mM 3, 3′-DTSSP for 30 min at 25°C. The reaction was stopped by adding Tris-HCl (pH 7.0) to a final concentration of 50 mM and incubated for 15 min at 25°C. Cross-linking was analyzed by non-reducing SDS-PAGE and immunoblotting with the purified AtGRIP antibody.

### Yeast Two-hybrid Analysis

All of the constructs in pGBKT7 and pGADT7 were used to transform AH109 yeast cells (Clontech) by using LiAc methods [29] to test self-interactions and interactions between AtGRIP_aa1-604_ and AtGRIP_aa605–788_. Colonies were selected on SD (synthetic drop-out medium)/-Trp/-Leu medium, and the selected cells were then streaked on SD/-Trp/-Leu/-His/-Ade plates to test their interactions. Positive yeast transformants were tested with X-gal (Clontech) to detect MEL1 reporter gene expression.

### Transient Expression of GFP and mCherry-fused Proteins in *Arabidopsis* Protoplasts

All of the constructs were expressed in *Arabidopsis* protoplasts by polyethylene glycol-mediated transformation using a protocol from Yoo et al. [30].

In brief, approximately 15 expanded leaves from 4-week-old plants with a shorter photoperiod were cut into 0.5–1 mm strips and incubated with 2 mL of enzyme solution [1% Cellulase R-10, 0.25% Macerozyme R-10, 400 mM mannitol, 20 mM KCl, 10 mM CaCl_2_, 20 mM 2-(N-morpholino) ethanesulfonic acid (MES) (KOH), pH 5.7 and 0.1% BSA] at 23°C for 3 h with gentle agitation (50 rpm). After incubation, the protoplast suspension was filtered through a Nylon mesh (50 µm), and the protoplasts were collected by centrifugation at 100 g for 1–2 min. The pelleted protoplasts were resuspended in ice-cold W5 buffer [154 mM NaCl, 125 mM CaCl_2_, 5 mM KCl and 2 mM MES (KOH), pH 5.7] and washed twice. After the second wash, the protoplasts were incubated on ice in W5 buffer for 30 min.

For DNA transformation, the protoplasts were collected by centrifugation at 100 g for 50 sec and resuspended in MMG buffer [400 mM mannitol, 15 mM MgCl_2_ and 4 mM MES (KOH), pH 5.7]. DNA (20–40 µg) was added to 200 µL of the protoplast suspension followed by 220 µL of PEG solution (40% PEG 4000, 200 mM mannitol and 100 mM CaCl_2_). The cells were mixed gently and incubated at 23°C for 30 min, and the mixture was then diluted with 800 µL of W5 buffer. The protoplasts were recovered by centrifugation at 100 g for 1–2 min, resuspended in 1 mL of W5 buffer and incubated at 23°C in the dark.

### Confocal Microscopy

All samples were observed by using a Zeiss LSM 510 META confocal laser-scanning microscope with a Zeiss 63× oil objective (NA 1.4) (Carl Zeiss Far East Co.). The excitation and emission wavelengths were 488 nm and 505–530 nm for GFP and 543 nm and 560–615 nm for mCherry. All images were obtained from single focal planes along the z-axis. The images were processed with Adobe Photoshop CS2 (Adobe Systems, San Jose, CA).

## Supporting Information

Figure S1 **Dimerization analysis of full-length AtGRIP, AtGRIP_aa711–788_, and AtGRIP_aa711–753_ by yeast two-hybrid assays.** (A) The interaction among full-length AtGRIP, AtGRIP (AA711–788) and AtGRIP (AA711–753) was analyzed by using the yeast two-hybrid system. After 7 days on synthetic plates lacking adenine, histidine, leucine and tryptophane at 30°C, only the combinations of pGBKT7-AtGRIP with pGADT7-AtGRIP (1), pGBKT7-AtGRIP (AA711–788) with pGADT7-AtGRIP (AA711–788) (3) produced colonies. pGBKT7-AtGRIP (AA711–753) with pGADT7-AtGRIP (AA711–753) did not generate colonie (2). (B) Colonies in (1) and (3) tested positive in the X-gal assay, demonstrating that these fusion proteins can respectively interact. Colonie in (2) did not test positive in the X-gal assay.
(TIF)
