# Supplementary figures and images for: The C-TERMINUS of AtGRIP Is Crucial for Its Self-Association and for Targeting to Golgi Stacks in Arabidopsis
Source: PLoS One. 2014 Jun 5;9(6):e98963. doi: 10.1371/journal.pone.0098963 (PMC4047078; doi:10.1371/journal.pone.0098963)

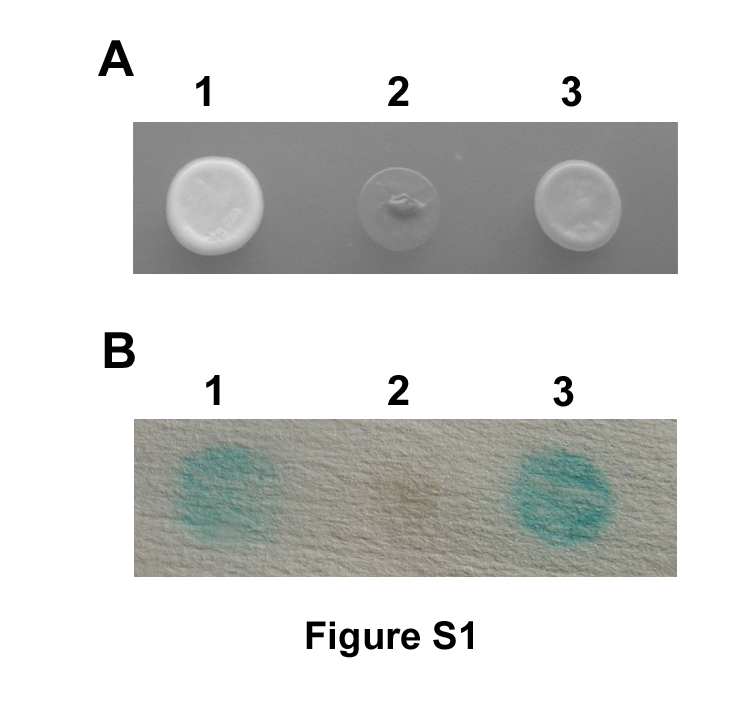

Supplement: Figure S1 — Dimerization analysis of full-length AtGRIP, AtGRIPaa711–788, and AtGRIPaa711–753 by yeast two-hybrid assays. (A) The interaction among full-length AtGRIP, AtGRIP (AA711–788) and AtGRIP (AA711–753) was analyzed by using the yeast two-hybrid system. After 7 days on synthetic plates lacking adenine, histidine, leucine and tryptophane at 30°C, only the combinations of pGBKT7-AtGRIP with pGADT7-AtGRIP (1), pGBKT7-AtGRIP (AA711–788) with pGADT7-AtGRIP (AA711–788) (3) produced colonies. pGBKT7-AtGRIP (AA711–753) with pGADT7-AtGRIP (AA711–753) did not generate colonie (2). (B) Colonies in (1) and (3) tested positive in the X-gal assay, demonstrating that these fusion proteins can respectively interact. Colonie in (2) did not test positive in the X-gal assay. (TIF) [file pone.0098963.s001.tif]
